# Effects of wasp venom on venous thrombosis in rats

**DOI:** 10.22038/IJBMS.2022.63219.13962

**Published:** 2022-07

**Authors:** Fan-mao Jin, Mei Wang, Xiu-mei Wu, Huai Xiao, De-xiao Wang, Guang-ming Wang, Cheng-gui Zhang, Hai-rong Zhao

**Affiliations:** 1 Lishui City People’s Hospital, Lishui, Zhejiang 323000, People’s Republic of China; 2 Yunnan Provincial Key Laboratory of Entomological Biopharmaceutical R&D, Dali University, Dali, China; 3 National-Local Joint Engineering Research Center of Entomoceutics, Dali University, Dali, China; 4 Genetic Testing Center, The First Affiliated Hospital of Dali University, Dali University, Dali, China; # These authors contributed equally to this work

**Keywords:** Argatroban, Blood coagulation factors, Platelet activation, Venous thrombosis, WV

## Abstract

**Objective(s):**

This study aimed to investigate the potential effects of wasp venom (WV) from *Vespa magnifica* on antithrombosis in rats with inferior vena cava (IVC) thrombosis.

**Materials and Methods:**

The thrombosis rat model was established by improving the IVC stenosis, in which rats were subjected to IVC ligation for 75 min. Rats were administered argatroban (IP) or WV (s.c.) for 4 hr after IVC thrombosis. The weight, inhibition rate, and pathological morphology of the thrombosis induced by IVC ligation and the variation in four coagulation parameters, coagulation factors, and CD61+CD62P+ were simultaneously determined in IVC rats.

**Results:**

The thrombus formed as a result of IVC ligation was stable. Compared with the control group, the weight of the thrombus was significantly reduced in the argatroban group. Thrombus weight was reduced by treatment with 0.6, 0.2, and 0.05 mg/kg WV, with inhibition rates of 52.19%, 35.32%, and 28.98%, respectively. Inflammatory cells adhered to and infiltrated the vessel wall in the IVC group more than in the sham group. However, the pathological morphology and CD61+CD62P+ of the WV treatment groups tended to be normal.

**Conclusion:**

We improved the model of IVC thrombosis to be suitable for evaluation of antithrombotic drugs. Our findings demonstrated that WV could inhibit IVC thrombosis associated with reducing coagulation factors V and CD61+CD62p expression in rats.

## Introduction

Deep venous thrombosis (DVT) is caused by slow blood flow, injury to vascular endothelial cells, and other factors that cause venous reflux disease through blood coagulation in the deep veins. The exfoliated thrombus may cause pulmonary embolism (PE). DVT and PE are collectively referred to as venous thromboembolism, and they are both manifestations of the same disease at different stages (1, 2). DVT, PE, and post-thrombus syndrome (PTS) seriously affect a patient’s quality of life and can even lead to death (3).

The primary treatment for DVT is anticoagulant therapy (4). However, the anticoagulants that are commonly used today carry a high risk of severe bleeding (5) and require international normalized ratio monitoring during the treatment course, which is inconvenient for patients (6). Therefore, new anticoagulant therapies are urgently needed. There are many methods for developing DVT animal models that can be used to investigate the preventive effect of anticoagulants (7-9). The current rat thrombosis model caused by inferior vena cava (IVC) ligation has been used to study the preventive effect of anticoagulants (10). However, therapeutic drug administration is currently being advocated in the study of pharmacodynamics of new anticoagulant drugs.

Wasp venom (WV) extracted from *Vespa magnifica* (Smith) represents a complex mixture of biologically active proteins and peptides, such as phospholipases, hyaluronidase, phosphatase, α-glucosidase, serotonin, histamine, dopamine, noradrenaline, and adrenaline, with significant pharmacological effects and biological activity. Our previous results showed that the WV extracted from *Vespa magnifica* Smith (Vespidae) had a protective effect on rheumatoid arthritis (11) and stroke (12) in rats. Our previous study has also confirmed that four compounds, including 5-hydroxytryptamine, vespakin M, mastoparan M, and vespid chemotactic peptide M were purified and identified from WV (13). In addition, vespakin M (14) and mastoparan M (15) protect neurons against cell death and axonal injury after ischemic stroke.

However, no study has yet reported the effects of WV on antithrombosis. In this study, a new, efficient, and stable venous thrombosis animal model suitable for therapeutic administration was established. In order to verify the adaptability of this improved model, argatroban, a direct thrombin inhibitor, was used as a positive control to demonstrate the stability and scientificity of this model. Following that, the effect of WV on antithrombosis was evaluated using this model.

## Materials and Methods

***Animals***

Adult male Sprague-Dawley (SD) rats weighing 260-280 g were obtained from Hunan Silaike Jingda Experimental Animal Co. LTD. Animal certificate No.: SCXK (xiang) k2017-0004. The animals were kept pathogen-free in a humidity- and temperature-controlled environment with a 12 hr light/dark cycle, with access to a standard diet and water *ad libitum*. All experimental procedures and animal housing in this study were designed and conducted in accordance with the approval of the Institutional Animal Care and Use Committee of Dali University, China (Animal ethics No.: DLU2017-0117).

***Animal Surgery***

**Figure 1:**
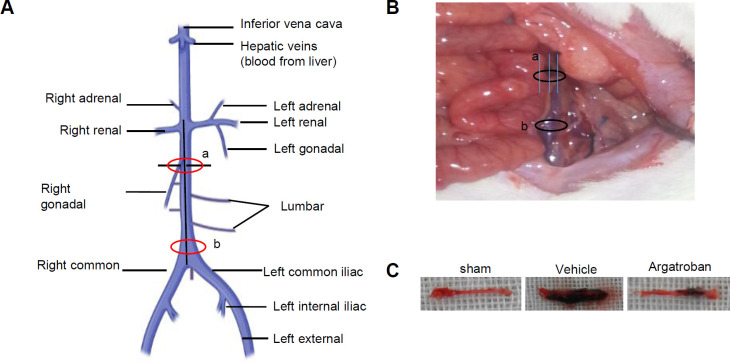
Construction and evaluation of the IVC thrombus model

The thrombosis model was constructed by “narrowing” the IVC to investigate the effect of anticoagulant prophylaxis. In order to establish a stable IVC thrombosis model to detect the antithrombotic activity of drugs, the thrombus model was improved according to the methods (16). The procedure was carried out as follows: rats were anesthetized with 5.0% isoflurane and maintained by inhalation of 1.5% isoflurane driven by 100% oxygen flow using the EZ-Anesthesia system (Euthanex Corp., Palmer, PA, USA) in a supine position. Laparotomy was then performed and the intestines were exteriorized with sterile saline to prevent drying during the whole procedure. IVC was fully exposed by midline laparotomy dissected at the level of the renal veins, then the side branches were ligated using a 4-0 suture. In addition, a 4-0 suture was passed 1 mm below the left renal veins, which was one of the ligation points in IVC. Meanwhile, three equal-length 2-0 lines were placed parallel to the IVC (Figure 1A (a)), ligated below the renal veins (Figure 1B (a)) using a 4-0 suture at a specific ligation point. Following that, the blood vessels at the IVC ligation points, from which the three equal-length 2-0 lines would be removed after 1.5 hr, were reduced from 80%–85% as the standard. Finally, the iliac vein (Figures 1A, B (a)) was tied with a slipknot using a 2-0 line. The surgical preparation is shown in Figures 1A and B. After 1.5 hr, the slipknot at the junction of the left and right common iliac veins (b) was unraveled, and the three 2-0 surgical lines were removed from the ligation point below the left renal vein. 

***Improved model evaluation and drug administration***

Argatroban (H20050918) was selected as the positive drug to evaluate the stability of the improved model. A total of 60 rats were randomly divided into six groups: a vehicle group and five argatroban groups (7.8, 5.5, 3.9, 2.8, and 2.0 mg/kg, IP). Next, 50 rats were randomly divided into five groups: a vehicle group, an argatroban group (5.5 mg/kg, IP), and WV (0.05, 0.2, and 0.6 mg/kg, SC) groups. WV was provided by the National-Local Joint Engineering Research Center of Entomoceutics, Dali, Yunnan. And the HPLC method was used for quality control of WV, the chromatographic conditions of which were as described previously. The animals were administered argatroban intravenously or WV subcutaneously twice, 4 hr and 24 hr after IVC ligation.

***Thrombus weight***

**Figure 2:**
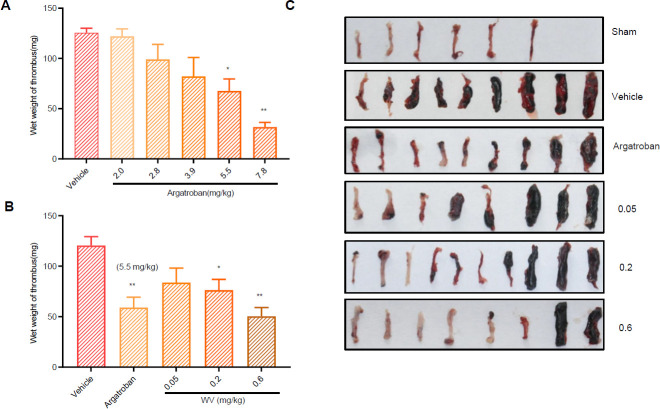
The effect of wasp venom on thrombus weight and appearance after inferior vena cava thrombosis in rats. (A) Thrombus weight was decreased in the argatroban groups (5.5 and 7.8 mg/kg) after inferior vena cava compared with the vehicle group,*P<0.05, **P<0.01, (n=8~10). (B) Thrombus wet weight was lower in the argatroban (5.5 and 7.8 mg/kg) groups (*P<*0.01, n=8~10) and the wasp venom (WV) groups (0.6 mg/kg, *P<*0.05; 0.2 mg/kg, *P<*0.01, n=8~10) according to thrombus weighing. (C) The appearances of the thrombi

All rats were anesthetized with 5.0% isoflurane and maintained by inhalation of 1.5% isoflurane driven by 100% v oxygen flow using the EZ-Anesthesia system after the second administration for 1 hr, and the IVC within their thrombus was removed and weighed, as seen in Figure 2.

***Four coagulation parameters and activity of coagulation factors II, V, and X***

Rats were anesthetized after IVC. Blood was collected from the abdominal aorta and mixed with 3.2% sodium citrate at a ratio of 1:9. Following that, the blood was centrifuged at 3,000 rpm for 10 min to separate the plasma. The plasma was analyzed using an automatic coagulation apparatus (CA1500).

***Histological assessment of the thrombus***

Hematoxylin-eosin staining was performed as previously described (15). Briefly, thrombus fragments were fixed with 10% formalin for 24 hr, washed, paraffin-embedded, and cut into 5 μm sections using a microtome (Leica, Germany). Inflammatory cell infiltration of the vascular wall of endothelial cells was observed by routine HE staining.

***Flow cytometry (FCM)***

We performed FCM staining for CD61-FITC (a marker of all platelets) and CD62P-PE (a marker of activated platelets) by whole blood sample. Single-cell suspensions were prepared and cell numbers were counted by a Coulter counter (Thermo Fisher). Cells were washed with buffer (PBS with 0.5% bovine serum albumin and 0.02% sodium azide) three times and subsequently stained with fluorochrome-conjugated monoclonal antibodies: CD61-PE (#561912, BD Biosciences ) and CD62P-FITC (#550866, BD Biosciences). Samples were analyzed using Flow Cytometry (CytoFLEX S). Subsequent analysis was performed with FlowJo software (Tree Star Inc., San Carlos, CA, USA). 

***Statistical analysis***

Statistical analysis was performed using the GraphPad Prism 7 software. Homogeneity of variance was examined using Levene’s test. If there was no statistical significance (*P*>0.05), one-way ANOVA was used; if ANOVA was statistically significant (*P*≤0.05), the LSD test (parametric method) was performed. The Kruskal-Wallis test was performed if the variance was not consistent (*P*<0.05). If the Kruskal-Wallis test displayed statistical significance (*P*<0.05), the Mann-Whitney method was used to make any pairwise comparison between the means.

## Results

***Improved IVC model was stable and argatroban decreases thrombus weight***

Our findings demonstrated that a stable thrombus can be formed 4 hr after IVC ligation (Figure 1C). The appearance of thrombus is shown in Figure 1C. Compared with the vehicle group, the thrombus wet weight was lower in the argatroban groups (5.5 and 7.8 mg/kg) (*P*<0.01) and showed dose-dependence (Figure 2A). Therefore, 5.5 mg/kg argatroban was selected as the positive control to determine the antithrombotic effect of WV (SC).

***WV reduces thrombus wet weight after IVC***

Low-velocity blood is a major cause of thrombosis. Because of the improved IVC ligation model, the trunk blood flow rate of the inferior vena cava was reduced from 80% to 85%, leading to activation of venous endothelial cells and initiation of exogenous coagulation pathways to induce thrombosis. The antithrombotic effects in the WV groups (0.2 mg/kg, *P*<0.05; 0.6 mg/kg, *P*<0.01) were determined by weighing the thrombus (Figure 2B) and observing its appearance (Figure 2C). The internal density of thrombus was more intensive in the vehicle group, whereas it was smaller and lighter in the other groups.

**Figure 3:**
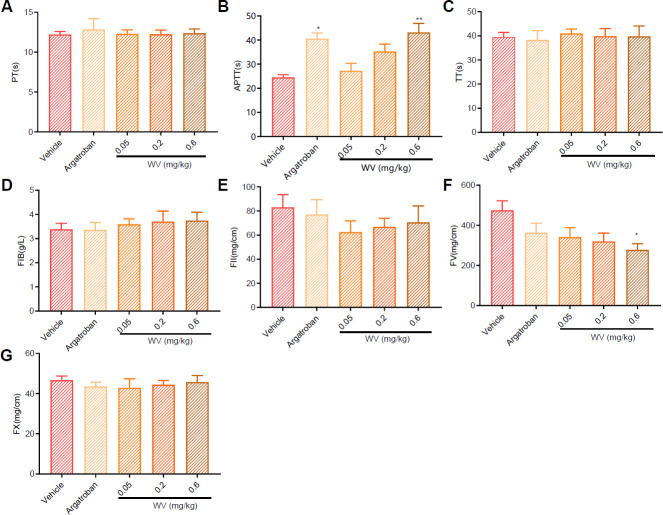
The effect of wasp venom (WV) on four coagulation parameters and coagulation factors II, V, and X after inferior vena cava (IVC) thrombosis in rats

**Figure 4:**
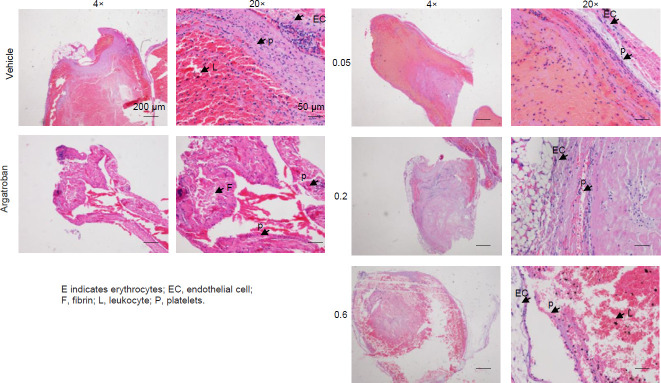
Histological evaluation of the ligated inferior vena cava (IVC). Thrombi were primarily composed of fibrin, platelets, and erythrocytes. Leukocytes were found on occasion within the thrombus. A large number of filamentous fibers and white blood cells were observed in the vein white thrombus

WV lengthens APTT and decreases coagulation factors V after IVC and are used to determine whether fibrinogen is congenital or acquired. Prothrombin, the defect of coagulation factors VII and X, and four coagulation parameters were examined after IVC thrombosis. There were no statistical differences in prothrombin time (PT) (Figure 3A), thrombin time (TT) (Figure 3C), and fibrinogen (FIB) (Figure 4D) between each group, suggesting that WV did not affect the external coagulation pathway. Compared with the vehicle group, the activated partial thromboplastin time (APTT) was increased in the argatroban (5.5 mg/kg, *P*<0.05) and WV (0.6 mg/kg, *P*<0.01) groups (Figure 3B), indicating that the anti-clotting effect of WV may be due to the influence of endogenous coagulation. The activation of coagulation factors II, V, and X was also examined; there was no significant increase in the content of coagulation factors II (Figure 3E) and X (Figure 3G). WV (0.6 mg/kg, *P*<0.01) reduced coagulation factor V compared with the vehicle group (Figure 3F).

***Pathological changes associated with venous thrombosis and WV decreased CD61+CD62p expression***

**Figure 5:**
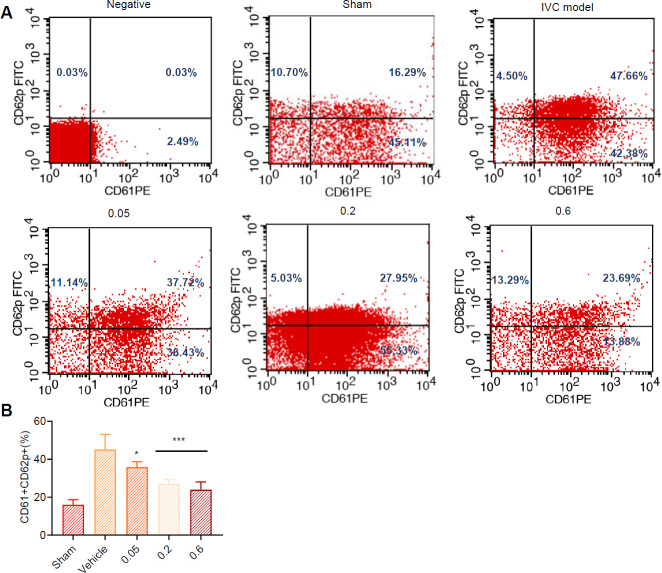
Effect of wasp venom (WV) on CD61 and CD62p expression. (A) Whole blood samples from inferior vena cava thrombus of rat were stained with CD61-PE and CD62p-FITC by FCM. Using the sham group lower expression of CD61+CD62p+ as negative control. (B) CD61+CD62p+ was quantized by FlowJo V10

According to morphological examination results, the thrombus mainly consisted of fibrin, platelets, and erythrocytes. Leukocytes (identified as monocytes and neutrophils based on their nuclear morphology) were also observed within the thrombi. Optical microscope observation revealed increased inflammatory cell infiltration in the vascular wall of the vehicle group compared with the sham group (Figure 4). In addition, intercellular spaces were infiltrated by inflammatory cells, and a white thrombus was visible in the vehicle group. However, argatroban (5.5 mg/kg) and WV (0.2 mg/kg, 0.6 mg/kg) treatment reduced inflammatory cell infiltration in the vascular wall, recanalized blood vessels, and decreased the thrombus. As seen in Figures 5A and 5B, WV decreased CD61+CD62p expression after IVC.

(A) Schematic diagram of IVC and its collateral veins. (B) Physical map of the inferior vena cava and its collateral vein anatomy (surgical technique). The red circle depicts the IVC ligation (a,b). Before ligation “a” with a 4-0 suture, three isometric 2-0 sutures parallel to the IVC were placed. Ligation of “b” with beaten knot using a 2-0 suture. After 1.5 hr, the junction of the left and right iliac vein “b” was unlocked, and ligation “a” three isometric 2-0 sutures were removed to reduce from 80% to 85% as the standard. (C) Evaluation of the IVC thrombus model with argatroban

## Discussion

Previous studies have shown that bee venom could increase in* vitro* and contain anti-coagulation factors, such as phospholipase A2 (PLA2) and melittin (17). WV or its compounds have higher cytotoxicity (18). Compared with bee venom, few pharmacological investigations have been undertaken for WV. In this study, an IVC thrombosis model in rats was successfully established by improving the “narrow method”, and WV could inhibit IVC thrombosis associated with reducing coagulation factors V and CD61+CD62p expression in rats. 

It is currently believed that DVT is caused by systemic stress due to surgical trauma and venous blood stasis **(**19). Activation of venous endothelial cells caused by blood stasis or hypoxia is the trigger for thrombosis (20, 21). Therefore, the initiation of venous thromboembolism is the activation of venous endothelial cells rather than direct injury (22). The thrombosis model established by “narrowing” the IVC (16) has previously been used to study the effect of anticoagulant prophylaxis. However, the thrombus produced by this method has poor stability. In comparison, we have made improvements to the model. In this study, the incomplete IVC ligation method (i.e., narrow method) was used to limit blood flow to induce thrombosis, and a rat IVC thrombosis model without mechanical destruction of the venous endothelium was established, which was consistent with clinical practice of venous thrombosis. Our findings demonstrated that a stable thrombus can be formed 4 hr after IVC ligation. The thrombosis was stable, and the animal survival rate was 98%. In addition, we confirmed WV reduced thrombus weight after IVC.

Argatroban, a potent, reversible thrombin inhibitor that affects coagulation factor II, directly inhibits coagulation factor A. Previous studies have shown (23, 24) that thrombin argatroban can be directly inactivated and combined with fibrin thrombus and thrombus directly into the internal. This significantly reduces the incidence of thrombosis without causing bleeding, and adverse reactions are reduced. Argatroban treatment of DVT and its complications in combination with conventional heparin treatment increased clinical efficacy and safety. However, previous domestic studies did not report a clear dose-response relationship. For the first time, an improved model of the inferior vena cava “stenosis method” was used to evaluate the efficacy of argatroban and WV. The dosage of argatroban negatively correlated with the size of the thrombus, and the weight of the thrombus gradually decreased with increasing dosage. The multiple test results were similar, indicating that the modeling method is reproducible, and the findings were consistent with previous studies (25). Therefore, the IVC model was successfully improved, and the effect of drug treatment was evaluated using this model.

According to previous studies (26, 27), endothelium dysfunction, activation of coagulation factor, decreased fibrinolysis function, and platelet activation all play important roles in the pathogenesis of thrombosis. The FX to fibrin formation process is known as a common approach. Prothrombin time (PT) and activated partial thromboplastin time (APTT) are used to determine whether fibrinogen is congenital or acquired. Prothrombin, the defect of coagulation factors VII and X, and fibrinogen (FIB) are the main proteins involved in the coagulation process. Fibrinogen reduction (<1.5 g/l) is observed in disseminated intravascular coagulation and primary fibrinolysis, severe hepatitis, and cirrhosis. Furthermore, it is used in the monitoring of snake venom (e.g., antithrombotic enzyme, defibrase) and thrombolytic therapy (UK, t-PA). Prolonged thrombin time was observed in anticoagulants containing increased heparin or were heparin-like. PT is involved in the exogenous coagulation pathway. In the current study, we confirmed WV lengthens APTT and decreases coagulation factors V after IVC, suggesting the anti-clotting effect of WV may be due to the influence of endogenous coagulation. Additionally, WV may contain compounds of anticoagulant factors V that require further study.

CD61, also known as platelet membrane glycoprotein 1B /III A, is the most abundant membrane glycoprotein on the surface of platelets and is the receptor that mediates the binding of platelets to fibrinogen. CD61 has become the main target of antithrombotic therapy and antiplatelet therapy due to its key role in thrombosis and platelet activation. CD62, also known as platelet activation-dependent particle surface membrane protein or P-selectin, is located in platelet A particle and endothelial rod tubular bodies and is the most specific molecular marker of platelet activation, mediating platelet rolling and adhesion on endothelial cells, and the interaction between platelets and monocytes. CD62P is exposed to platelet membrane after platelet activation and is involved in platelet adhesion to leukocytes as a receptor for p-soragglutinin glycoprotein ligand-1 (PSGL-1D). Our results demonstrated that WV decreased CD61+CD62p+ expression, suggesting platelet activity was blunted.

## Conclusion

We improved the IVC model to be suitable for evaluation of antithrombotic drugs. Our findings demonstrated that WV could inhibit IVC thrombosis associated with reducing coagulation factors V and CD61+CD62p expression in rats.

## Authors’ Contributions

ZHR and ZCG designed the experiments; JFM, WM, WDX, and WXM performed experiments and collected data; XH discussed the results and strategy; ZCG and ZHR supervised, directed, and managed the study; ZHR and ZCG approved the final version to be published.

## Conflicts of Interest

No conflict of interest exists in this study.
